# Machine Learning in Bioelectrocatalysis

**DOI:** 10.1002/advs.202306583

**Published:** 2023-11-09

**Authors:** Jiamin Huang, Yang Gao, Yanhong Chang, Jiajie Peng, Yadong Yu, Bin Wang

**Affiliations:** ^1^ Department of Environmental Science and Engineering University of Science and Technology Beijing Beijing 100083 China; ^2^ CAS Key Laboratory of Nanosystem and Hierarchical Fabrication National Center for Nanoscience and Technology Beijing 100190 China; ^3^ School of Computer Science Northwestern Polytechnical University Xi'an 710072 China; ^4^ College of Biotechnology and Pharmaceutical Engineering Nanjing Tech University Nanjing 211816 China

**Keywords:** bioelectrocatalysis, biosensors, interdisciplinary research, machine learning, microbial fuel cells

## Abstract

At present, the global energy crisis and environmental pollution coexist, and the demand for sustainable clean energy has been highly concerned. Bioelectrocatalysis that combines the benefits of biocatalysis and electrocatalysis produces high‐value chemicals, clean biofuel, and biodegradable new materials. It has been applied in biosensors, biofuel cells, and bioelectrosynthesis. However, there are certain flaws in the application process of bioelectrocatalysis, such as low accuracy/efficiency, poor stability, and limited experimental conditions. These issues can possibly be solved using machine learning (ML) in recent reports although the combination of them is still not mature. To summarize the progress of ML in bioelectrocatalysis, this paper first introduces the modeling process of ML, then focuses on the reports of ML in bioelectrocatalysis, and ultimately makes a summary and outlook about current issues and future directions. It is believed that there is plenty of scope for this interdisciplinary research direction.

## Graphical abstract

Bioelectrocatalysis for clean energy production and organic waste/environmental pollutant treatment has received a great deal of attention from scientists and engineers around the world. The introduction of machine learning (ML) in this field has just started. At present, ML is mainly applied to electrochemical biosensors and microbial fuel cells, and there are already visible achievements.

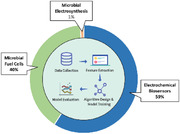

## 1 Introduction

The desire for sustainable and clean energy has sparked widespread interest due to the environmental issue created by the depletion of fossil fuel supplies and the production of greenhouse gases.^[^ ^1^ ^]^ For the production of high‐value chemicals, clean biofuels, and degradable new materials, bioelectrocatalysis offers a green, sustainable, and efficient selection.^[^ ^2^ ^]^ Bioelectrocatalysis employs biological system materials as catalysts to catalyze redox reactions on the electrode.^[^ ^3^ ^]^ It is a cross‐field of biocatalysis and electrocatalysis and fully exploits the benefits of mild biocatalysis conditions and low temperature,^[^ ^4^ ^]^ as well as the flexible conversion of electrical to chemical energy.^[^ ^5^ ^]^ Electrochemical reactions in the biocatalysis process safely deliver redox equivalents required for biocatalysis while consuming electricity provided by renewable resources.^[^ ^2^ ^]^

The basic functional portion in a bioelectrochemical system is a bioelectrocatalyst,^[^ ^5^ ^]^ which consists primarily of electroactive microbial cells and oxidoreductase.^[^ ^2^ ^]^ In 1912, Porter proposed to use intact living cells as biocatalysts for bioelectrocatalysis.^[^ ^6^ ^]^ In the 1960s, electrochemists expanded bioelectrocatalysts to isolated oxidoreductases.^[^ ^7^ ^]^ Subsequently, oxidoreductase and electroactive microbial cells were used to trigger enzyme fuel cells and microbial fuel cells (MFCs), respectively.^[^ ^5^ ^]^ Furthermore, electrochemical enzyme/microbial biosensors and enzyme/microbial electrosynthesis are catalyzed by oxidoreductase and electroactive microbial cells.^[^ ^8^ ^]^ Generally speaking, bioelectrocatalysis technologies have mainly been applied in biosensors, biofuel cells, and bioelectrosynthesis.

Machine learning (ML) demonstrates a “learning” experience related to artificial intelligence, and it learns and enhances its analysis by applying computing algorithms.^[^ ^9^ ^]^ As a significant subfield of artificial intelligence, ML has been widely used in image analysis, medical diagnosis, network intrusion detection and prediction, and other fields,^[^ ^10^ ^]^ indicating its ability to solve complex problems. Bioelectrocatalysis contains various influencing factors and complex interactions, which are far from the capability of simple controlled experiments. Therefore, ML has been introduced in recent years to analyze complex problems in different subfields of bioelectrocatalysis. For example, ML overcomes the low accuracy issue of traditional methods in biosensors and transforms ordinary biosensors into intelligent biosensors based on decision‐making systems to automatically predict the type or concentration of analytes.^[^ ^11^ ^]^ ML optimizes the efficiency of MFCs by a low‐cost approach in biofuel cells and thus compensates for the flaw that constrained laboratory circumstances cannot accurately represent the actual situation.^[^ ^12^ ^]^ For bioelectrosynthesis, it is a development prospect to maximumly introduce protein engineering in enzyme electrosynthesis and combine microbial electrosynthesis with synthetic biology.^[^ ^5^ ^]^ Although only several works have applied ML in bioelectrosynthesis, it has the potential to boost oxidoreductase and electroactive microbial cell activity. At present, there is progress in the emerging field of bioelectrocatalysis combined with ML, but there is a lack of review to systematically summarize the development of this field.

This review discusses the research progress of ML in bioelectrocatalysis, including a brief introduction to the ML modeling process for readers who are not majoring in computer sciences and ML applications in bioelectrocatalysis. Based on previous research, the applications of ML in electrochemical (EC) biosensors, MFCs, and microbial electrosynthesis are outlined. Finally, the current problems and prospects of ML in bioelectrocatalysis applications are discussed.

## 2 Machine Learning Modeling

**Figure 1:**
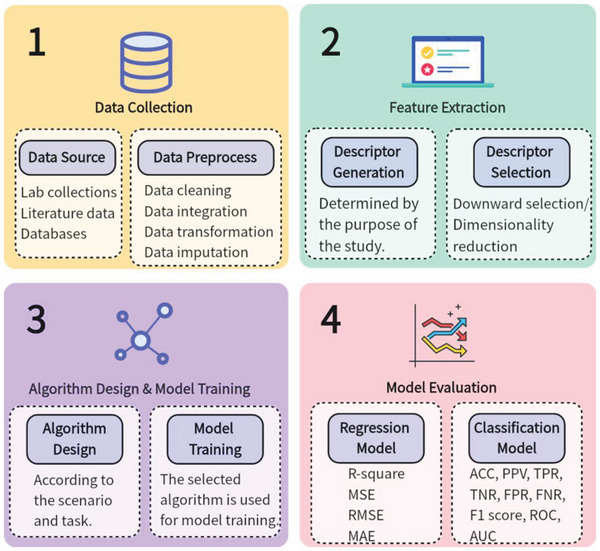
Workflow of building a traditional ML model. MSE: mean square error. RMSE: root‐mean‐square error. MAE: mean average error. ACC: accuracy. PPV: positive predictive value. TPR: true positive rate. TNR: true negative rate. FPR: false positive rate. FNR: false negative rate. ROC: receiver's operating characteristic. AUC: area under the receiver's operating characteristic.

Traditional ML modeling workflow consists mostly of data collection, feature extraction, algorithm design and model training, and model evaluation.^[^ ^13^ ^]^ The main procedures in developing an ML model are illustrated in **Figure** **1**: gathering data to generate a training dataset, generating and choosing mathematical descriptors, selecting a suitable algorithm and establishing the model, and assessing the model's quality and predicting abilities.^[^ ^13^ ^]^ It should be noted that the recently developed ML method, named deep learning, combines feature extraction and algorithm design to generate an end‐to‐end model.^[^ ^14^ ^]^

Here are the steps of traditional ML modeling. The first step is data collection. It is the most critical and time‐consuming process in the whole workflow, and a significant quantity of high‐quality datasets is a prerequisite to guaranteeing reliable prediction.^[^ ^15^ ^]^ The usual approach for creating datasets is to preprocess and clean the original data gathered through experiments or computations, and then encode them into binary values that computers can identify.^[^ ^16^ ^]^ The second step is feature extraction, including descriptor generation and selection. The third step is algorithm design and model training. Different algorithms perform differently on the same dataset at times. Linear regression, for example, is appropriate for datasets with linear relationships, but it frequently produces inadequate model performance when applied to datasets with nonlinear relationships.^[^ ^16^ ^]^ Besides, the selection and optimization of hyperparameters affect ML prediction performance.^[^ ^17^ ^]^ The fourth step is model evaluation, which employs various parameters to assess the regression and classification models. These steps are detailed in the sections that follow.

### 2.1 Data Collection

Data on the subject of bioelectrocatalysis are primarily gathered from experiments and literature, and sometimes data about the genome are required in this field when analyzing microbes, which is obtained from the National Center for Biotechnology Information (NCBI). The following are some specific examples.

*Data from Experiments*: Shabani et al.^[^ ^18^ ^]^ used support vector regression to determine the association between MFC output voltage and chemical oxygen demand (COD). The data are from experiments that yielded a collection of 48 data points (4 MFC, 4 COD values, each repeated three times). Each repetition signifies that a batch of water is supplied to the MFC, resulting in the output voltage peak. In another case, Fang et al.^[^ ^19^ ^]^ took experimental data as a training set and verification set. They established the relationship of four operating conditions with Coulombic efficiency (CE) and power density.

*Data from Literature*: The dataset used by Cai et al.^[^ ^20^ ^]^ contained 69 samples of microbial community data from different laboratory‐scale experiments,^[^ ^21^ ^]^ including 36 samples for acetate feed, 27 samples for wastewater feed, and 6 samples for carbohydrate feed, all of which were combined with ML techniques to predict feed substrates in MFCs.

*Data from NCBI*: Lesnik et al.^[^ ^22^ ^]^ created an ML model based on genomic data and tested its capacity to predict the resistance and resilience of MFCs. The genome dataset is stored in the NCBI sequence reading file. It includes 1810 amplicon sequence variants (ASVs) and was used as an input to the resistance and resilience classification model. ASV is a specific region in a DNA sequence that contains some variations associated with a specific gene or disease. Using this dataset, it was possible to verify that the decline in the accuracy of the elasticity model may be because elasticity is the product of a more complex interaction involving several genera, including those outside the assumption that it is indeed a potential stability indicator.

The quantity and quality of the original datasets influence the maximum level of ML performance. Noise data should be minimized and unbiased sampling should be ensured as much as feasible while building the first dataset for the ML model.^[^ ^16^, ^23^ ^]^ Moreover, in the process of collecting data about bioelectrocatalysis in the future, text extraction methods based on ML or currently popular large language models (LLMs) should also be considered to obtain data quickly.

### 2.2 Feature Extraction

The process of transforming original data into an algorithm is called characterization or feature extraction.^[^ ^24^ ^]^ It contains two steps, namely, descriptor creation and descriptor selection, both of which determine the quality and interpretability of a model.^[^ ^13^ ^]^ The chosen descriptors should have clearly defined chemical or physical meanings to effectively define the main characteristics and properties of data, and involve the least amount of computing work.^[^ ^16^ ^]^ It should be noted that data attributes dictate the top limit of maximum likelihood, whereas the algorithm only brings the model as near to the upper limit as is feasible.^[^ ^25^ ^]^

#### 2.2.1 Descriptor Generation

A good descriptor separates objects in the data space and encodes features linked to the modeled and predicted qualities.^[^ ^24^, ^26^ ^]^ Although context determines how a descriptor is generated, there are certain universal guidelines.^[^ ^13^ ^]^ First and foremost, the descriptor sets must give unique information. Second, descriptors should not be excessive. Redundant descriptors have a very low correlation with the modeled characteristics, and their values do not move much in the dataset (namely, low variance).^[^ ^13^ ^]^ Therefore, they should be deleted to avoid over‐fitting the model and damaging its ability to predict new data attributes.

In the field of bioelectrocatalysis, descriptors are associated with research purposes. For example, research on the stability analysis of cyclic voltammograms (CV)^[^ ^27^ ^]^ takes the cumulative voltage variance, cumulative current variance, and product as descriptors. Research on the relationship between MFC output voltage and COD^[^ ^28^ ^]^ takes the maximum peak height (PH), peak area (PA), peak duration (PD), acceleration rate (AR), and sedimentation rate (SR) as descriptors. The descriptors in different kinds of research differ significantly, so it is necessary to summarize descriptors in bioelectrocatalysis.

#### 2.2.2 Descriptor Selection

There are two main strategies for descriptor selection: downward selection and dimensionality reduction.^[^ ^13^ ^]^ For downward selection, several statistical techniques are employed to condense a huge number of descriptors into a manageable quantity. An L1 regularization term is inserted in a regression model, and items with low correlation with the model are penalized by reducing them to zero, which is known as the least absolute shrinkage and selection operator (LASSO).^[^ ^29^ ^]^ After training, each descriptor's relevance is frequently assessed using a tree algorithm like the random forest.^[^ ^30^ ^]^ Dimensionality reduction is another method, and new descriptors are created by linearly combining the existing descriptors, in which principal component analysis (PCA) is the most popular method.^[^ ^31^ ^]^ PCA establishes the main components or a set of orthogonal vectors as new descriptors to speed up the construction of the ML model,^[^ ^32^ ^]^ but it might also lose some important information from data points.

Understanding the significance of descriptors is helpful for the initial feature extraction screening. Using Pearson correlation analysis, one may determine the significance of descriptors.^[^ ^33^ ^]^ For example, Shabani et al.^[^ ^18^ ^]^ extracted five characteristics from each peak: PH, PA, PD, AR, and SR. Then, the dimension of the dataset is reduced by deleting characteristics with low connection to the Pearson correlation coefficient (PCC) between features and COD values, and the number of features is decreased from 5 to 3 (PH, PA, PD).

### 2.3 Algorithm Design and Model Training

**Figure 2:**
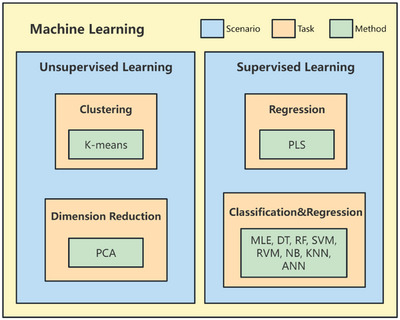
ML algorithms used in bioelectrocatalysis research. Unsupervised learning algorithms include k‐means and PCA, while supervised learning algorithms include decision tree (DT), support vector machine (SVM), relevance vector machine (RVM), naive Bayes (NB), k‐nearest neighbor (KNN), random forest (RF), maximum likelihood estimation (MLE), partial least squares (PLS) and artificial neural network (ANN). Algorithms should be selected according to research purposes. Some supervised learning algorithms are applied to both regression and classification.

After determining the optimal feature subset, algorithm design and model training are carried out. The first step is algorithm design. **Figure** **2** shows the reported ML algorithms used in bioelectrocatalysis. ML mainly includes unsupervised learning and supervised learning.^[^ ^34^ ^]^ Supervised learning refers to fitting a model to marked data (or a subset of data), in which there are some basic truth attributes, which are usually measured by experiments or assigned by researchers.^[^ ^35^ ^]^ In contrast, unsupervised learning identifies patterns in unlabeled data without providing basic truth information to the system in the form of predetermined tags.^[^ ^35^ ^]^ Unsupervised learning algorithms include clustering algorithms and dimension reduction algorithms. A set of data is clustered when its components are similar to one another,^[^ ^13^ ^]^ and the structure of data is understood by combining similar observations.^[^ ^36^ ^]^ Dimension reduction is to transform data with a large number of attributes (or dimensions) into low‐dimensional forms while preserving the different relationships between data points^[^ ^35^ ^]^ for data visualization and deleting features without information.^[^ ^36^ ^]^ Supervised learning algorithm includes classification algorithm and regression algorithm. Classification means that the output value is classified (assigning data to many categories), and regression means that the output value is digital (a continuous set of values).^[^ ^36^ ^]^

The second step is model training. Typically, the available data is split into a training set, a verification set, and a test set.^[^ ^35^ ^]^ The training set is used directly to train and build different models, the verification set is used to monitor training and select algorithms and hyperparameters, and the test set is used to evaluate the model's performance and predict errors on unused test datasets during training.^[^ ^35^, ^36^ ^]^

### 2.4 Model Evaluation

A high‐performing ML model should be able to predict unknown data as well as to fit existing data.^[^ ^16^ ^]^ The coefficient of determination (R^2^),^[^ ^19^, ^37^ ^]^ mean square error (MSE),^[^ ^12^, ^18^, ^37^, ^38^ ^]^ root‐mean‐square error (RMSE)^[^ ^19^, ^22^, ^37^, ^38^, ^39^ ^]^ and mean average error (MAE)^[^ ^22^, ^38^ ^]^ are commonly used to assess the predictive accuracy of regression models. To examine the variations in prediction performance across different categorization models, accuracy, precision, recall, specificity, and F1 score are commonly utilized.^[^ ^40^ ^]^ Furthermore, the receiver's operating characteristic (ROC) curve is an essential metric for evaluating the performance of a non‐uniformly distributed sample classifier,^[^ ^41^ ^]^ with the area under the ROC curve (AUC) quantitatively representing the model's prediction performance.^[^ ^37^, ^42^ ^]^

#### 2.4.1 Evaluation of Regression Model

For a dataset of size n, the real value *y_i_* (*i* = 1, 2, 3, …, n), average value $\bar{y}$, and the predicted value $\hat{y_{i}}\;$(*i* = 1, 2, 3, …, n) can be used to calculate the evaluation parameters of the regression model, mainly including R^2^, MSE, RMSE, and MAE. The defining characteristics and formulas of these parameters are shown in **Table** **1**.

**Table 1:** Evaluation parameters of the regression model.

Parameter	Definition	Characteristic	Formula^[^ ^13^, ^43^ ^]^
R^2^	Reflect the proportion of dependent variable changes that can be explained by independent variables using a regression relationship.	The normal range is [0,1], and the closer the model is to 1, the better it matches the data.	$\;R^{2}=\;1-\;\frac{\sum_{i\;=\;1}^{n}{\left(y_{i}-\hat{y_{i}}\right)}^{2}}{\sum_{i\;=\;1}^{n}{\left(y_{i}-\bar{y}\right)}^{2}}=\frac{\sum_{i=1}^{n}{\left(\hat{y_{i}}-\bar{y}\right)}^{2}}{\sum_{i=1}^{n}{\left(y_{i}-\bar{y}\right)}^{2}}\;$
MSE	It is often used to detect the difference between the model‘s predicted and actual values.	The degree of data change may be calculated, and the lower the number, the more accurate the prediction model is at representing the experimental data.	$MSE\;=\frac{1}{n}\;\sum_{i\;=\;1}^{n}{\left(y_{i}-\hat{y_{i}}\right)}^{2}$
RMSE	The square root is used to calculate the difference between the predicted and actual values based on MSE.	It is an order of magnitude with data, and it is easier to perceive data, but it is susceptible to outliers.	$RMSE\;=\sqrt{\frac{\sum_{i=1}^{n}{\left(\hat{y_{i}}-y_{i}\right)}^{2}}{n}}\;$
MAE	The mean difference between the predicted and actual values.	It reflects the actual situation of prediction error.	$MAE\;=\frac{1}{n}\;\sum_{i\;=\;1}^{n}\left|y_{i}-\hat{y_{i}}\right|$

In bioelectrocatalysis, R^2^ is a commonly used evaluation index to judge the accuracy of the model prediction. For example, in the study of Vakilian et al.,^[^ ^37h^ ^]^ R^2^ of different ML algorithms for predicting nitrate concentration was compared. Results show that the R^2^ of SVM (0.97 and 0.96 for the prediction of nitrate concentration of plant enzyme and bacterial enzyme, respectively) was higher than DT, NB, RF, ANN, and least‐square support vector machine (LSSVM), indicating its good prediction accuracy and model performance.

#### 2.4.2 Evaluation of Classification Model

**Figure 3:**
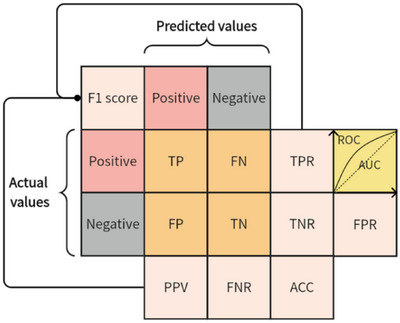
Confusion matrix metrics and classification model evaluation methods.

The confusion matrix is a common format for conveying accuracy evaluation in the form of an n‐by‐n matrix. The four types of confusion matrix metrics are as follows^[^ ^40^ ^]^: (1) True Positives (TP): The number of samples with positive predictive values and positive true values. (2) False Negatives (FN): The number of samples with negative predictive values and positive true values. (3) False Positives (FP): The number of samples with positive predictive values and negative true values. (4) True Negatives (TN): The number of samples with negative predictive values and negative true values. Confusion matrix metrics and classification model evaluation methods are shown in **Figure** **3**, and the definitions and formulas of the related assessment parameters are shown in **Table** **2**.

**Table 2:** Correlation evaluation parameters of the confusion matrix.

Parameter	Definition	Formula^[^ ^36^, ^40^ ^]^
ACC	The proportion of all instances predicted correctly.	$ACC\;=\frac{TP+TN}{TP+TN+FP+FN}\;$
PPV	The proportion of instances that are predicted to be positive and turned out to be positive.	$PPV\;=\frac{TP}{TP+FP}\;$
TPR	The proportion of instances predicted to be positive and actually positive to all positive instances.	$TPR\;=\frac{TP}{TP+FN}\;$
TNR	The proportion of instances that were predicted to be negative and were actually negative to all negative instances.	$TNR\;=\frac{TN}{TN+FP}\;$
FPR	The proportion of instances that are predicted to be positive and actually negative to all negative instances.	$FPR\;=\frac{FP}{FP+TN}\;$
FNR	The proportion of instances that are predicted to be negative and actually positive to all positive instances.	$FNR\;=\frac{FN}{TN+FN}\;$
F1 score	Harmonic average of recall and accuracy.	$F1\;score\;=\frac{2\cdot TP}{2\cdot TP+FP+FN}\;=\;2\cdot \frac{PPV\cdot TPR}{PPV+TPR}$

Circumstances where the model rightly predicts negative/positive classes are referred to as TN/TP, whereas circumstances where the model wrongly predicts negative/positive classes are referred to as FN/FP. The confusion matrix is used to calculate several assessment parameters, including accuracy (ACC), precision (also known as positive predictive value (PPV)), recall (also called true positive rate (TPR)), specificity (also called true negative rate (TNR), false positive rate (FPR) and false negative rate (FNR), and F1 score.

TPR and FPR in Table 2 represent the vertical and horizontal axes of the ROC curve, respectively. Different points are formed progressively by continually increasing the classification threshold, and these points are eventually joined to form a ROC curve. The vertical axis TPR in ROC represents the proportion where the outcome is positive and the prediction is also positive. Therefore, the higher the model prediction performance, the closer the ROC curve is to the upper left corner.^[^ ^44^ ^]^ If the ROC curves of the two models cross each other, it is difficult to conclude intuitively, then AUC is calculated to compare the models. When the AUC value is 1, it means that the model gets a perfect prediction no matter what threshold is set. When the AUC value is in the range of 0.5–1, it means that the model is superior to a random guess, and it has a predictive value if the threshold is set properly. When the AUC value is 0.5, it means that the model has no predictive value like a random guess.

In bioelectrocatalysis, PPV, TPR, and F1 score are often used to evaluate classification models. Ganguly et al.^[^ ^45^ ^]^ used multiplexed point of care (POC) biosensors to classify disease states based on severity, in which an RF model was used for digital classification. In the “infectious, systemic” state, PPV, TPR, and F1 score exhibited their highest values. This is desirable because this state corresponds to the peak of disease and entails the widespread dissemination of disease‐causing microorganisms throughout the body, as evidenced by elevated levels of all targeted inflammatory biomarkers in urine.

### 2.5 Summary of Machine Learning Modeling

ML modeling mainly includes four steps: data collection, feature extraction, algorithm design, model training, and model evaluation. Data collection is the foundation of model building, and both the quantity and quality of data sets are critical. Feature extraction transforms raw data into algorithms, including descriptor generation and descriptor selection. The selected descriptor should involve the least computational effort. Algorithm design and model training are the main parts of ML modeling, and selecting the right algorithm is the key step. Model evaluation is the evaluation of the ML modeling effect. There are different parameters to evaluate regression and classification models.

## 3 Applications of Machine Learning in Bioelectrocatalysis

**Figure 4:**
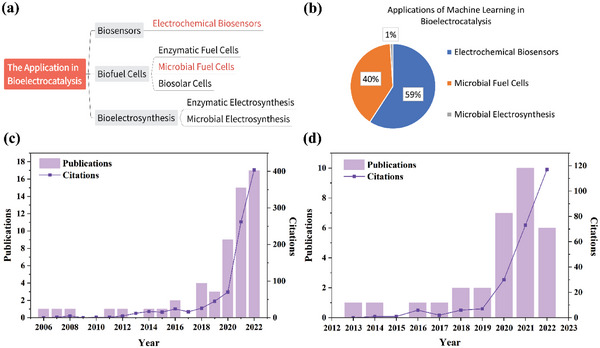
a) Classification of bioelectrocatalytic applications. b) Percentage of ML applications in bioelectrocatalysis. c) The number of publications and citations by year on WOS for the topic terms “Machine Learning” and “Electrochemical Biosensors”. d) The number of publications and citations by year on WOS for the topic terms “Machine Learning” and “Microbial Fuel Cells”.

The applications of bioelectrocatalysis mainly include biosensors, biofuel cells, and bioelectrosynthesis, and in red font, the areas where ML is widely applied are electrochemical (EC) biosensors and MFCs (**Figure** **4**a). To the best of our knowledge, ML was first applied to bioelectrocatalysis in 2006, and the specific application is an EC biosensor.^[^ ^46^ ^]^ In 2013, ML was applied to MFCs,^[^ ^19^ ^]^ and microbial electrosynthesis^[^ ^47^ ^]^ in 2020. By searching the papers published till December 31, 2022, on Web of Science (WOS) using “Machine Learning” and one additional keyword like “Electrochemical Biosensors”, “Microbial Fuel Cells”, “Enzymatic Fuel Cells”, “Microbial Electrosynthesis”, “Biosolar Cells”, or “Enzymatic Electrosynthesis”, the number of publications found after manual screening and supplementation is shown in **Table** **3** and Figure 4b. The number of publications and citations by topic terms “Machine Learning” and “Electrochemical Biosensors” on WOS is shown in Figure 4c, while the information for “Machine Learning” and “Microbial Fuel Cells” is in Figure 4d. It is clear that the attention to these topics increased rapidly after 2019, and more were paid to the use of ML in EC biosensors. Moreover, it is found the top three ML algorithms applied in bioelectrocatalysis are ANN (40 times), PCA (33 times), and SVM (14 times). The following sections discuss the applications of different ML algorithms in EC biosensors, MFCs, and microbial electrosynthesis.

**Table 3:** The number of publications on ML for bioelectrocatalytic applications.

Topic words		Number of publications on WOS	Number of publications on WOS (after manual screening)	Number of manual additions to the literature	Total number of literature
Machine Learning+	Electrochemical Biosensors	58	24	31	55
	Microbial Fuel Cells	31	11	26	37
	Enzymatic Fuel Cells	1	0	0	0
	Microbial Electrosynthesis	1	0	0	1
	Biosolar Cells	0	0	0	0
	Enzymatic Electrosynthesis	0	0	0	0

### 3.1 Applications of Machine Learning in Electrochemical Biosensors

Analytical devices that provide information about biological processes through sensors are called biosensors.^[^ ^48^ ^]^ More specifically, EC biosensors are a type of biosensors that combine a recognition element and an electronic transducer to detect analytes in body fluids with high sensitivity.^[^ ^49^ ^]^ The performance of biosensors is usually impacted by impurities, and ML assists in removing the signal acquired from pollutants to gain high sensitivity.^[^ ^48^ ^]^ By incorporating ML into biosensing systems, data interpretation also becomes simpler and more effective.^[^ ^37^, ^50^ ^]^ ML was reported to efficiently manage vast amounts of sensing data with complicated matrices or samples and directly, automatically, precisely, and swiftly help biosensors read out findings, which may then be utilized to create better biosensors.^[^ ^11^ ^]^ **Table** **4** shows the applications of different ML algorithms in EC biosensors from 2018 to 2022 (in descending order of publication date) from 16 research papers.

**Table 4:** Applications of ML in EC biosensors.

#	Algorithms	Application Scenarios	Advantages	Disadvantages	Results	Ref.
1	PCA	Cluster and classify the sugar content from the amperometric microbial biosensor.	No additional sample processing is required. Fast measurement.	—	Data variance percentages of 92.80% and 89.40% for the two main components.	[51]
2	BPNN^a)^	Identify and predict glucose and lactate concentrations from the nonenzymatic EC biosensor.	High selectivity, high sensitivity, and wide range of detection.	The data collection process is time‐consuming and expensive.	R^b)^: 0.9997, RSD^c)^: less than 6.5%.	[38b]
3	RF	Multiplexed point of care biosensor to implement severity‐based disease state stratification.	Solve the problem of bias and high variance of DT. Adding new data will not be affected much.	The accuracy is not high.	The accuracy was 70.88%.	[45]
4	PCA	Cluster and classify the type and concentration of pesticides from the clay/AuNPs/AChE^d)^ biosensor.	Effective determination of pesticide types and their corresponding concentrations.	Expensive and complex in some issues.	The total concentration of the pesticide mixture in the actual sample was 0.5 ng mL^−1^ for a particularly low identification.	[52]
5	RF	Apply to ultrasensitive combinatorial EC urine biosensor to implement severity‐based disease state stratification.	Solved the problem of bias and high variance of DT. Adding new data will not be affected much.	Only the 3 most important features were studied.	The accuracy was 98.437%.	[53]
6	PCA, PLS‐DA^e)^, SISSO^f)^	Apply to modular label‐free EC biosensor to make the COVID‐19 screening into healthy and infected groups.	Compared to PLS‐DA, the SISSO‐based learning task provides simpler descriptors.	Lack of further analysis of large samples.	The accuracy was 100%.	[54]
7	PCA	Determine the main contributors of variation in the BES^g)^ signals.	Simple and effective.	Not applicable to nonlinear cases.	The key contributors are pH, VFA^h)^ concentration, and temperature.	[55]
8	SVD^i)^, PCR^j)^	SVD: analyze the MTX^k)^ concentration from EIS^l)^, PCR: analyze the correlation between theoretical and measured concentrations of MTX.	SVD enables easier analysis of capacitance changes.	—	The R^2^ values of two capacitive biosensors measuring MTX concentration were obtained by PCR as 0.99.	[56]
9	SVR, ANN	Establish the Ca^2+^ concentration processing model.	SVR: Obtain the global optimal solution. High reliability of prediction.	ANN: lack of learning, overfitting, and trouble figuring out the network‘s structure.	The EC biosensor could measure Ca^2+^ content in the range of 7.5–1000 m with a detection limit of 5.48 µm for the optimum algorithm of SVR.	[57]
10	PCA, SVR	Establish relationships between multiple barrier parameters and bacterial concentrations.	Accurately predict *E. coli* concentrations.	SVR: solely demonstrates the reliability of detecting *E. coli* concentrations at specified discrete levels.	The average training error and the average prediction error of SVR (*n* = 10) were 1.44 ± 0.052% and 1.52 ± 0.136%, respectively.	[58]
11	ANN	Analyze carbendazim residues in tea and rice.	Wide range of applications. No need to study the relationship between processing parameters.	Subjective choices may lead to overfitting or underfitting.	ANN has a larger R^2^ and smaller RMSE and has better performance than all traditional regression models.	[59]
12	PCA, SOM^m)^	Classify 31 wine samples from amperometric biosensors.	The SOM treatment provides a nice resolution.	The resolution of PCA is low.	The visualization of the output data based on nonlinear SOM is mostly consistent with linear PCA, enabling the differentiation of wines.	[60]
13	SVM	The decline in enzyme activity over time was used to predict nitrate levels.	Simple and gives the user a better knowledge of the system's behavior.	The kernel function's performance is affected by the type of sample dispersion in the feature space.	The R^2^ and MSE were 0.93 and 0.0016, respectively.	[37g]
14	LDA^n)^, MLE, BPNN	Classify pathogens from disposable all‐printed electronic biosensors.	Simple, fast, accurate, and economical.	MLE: computationally expensive, a waste of time. BPNN: when dealing with ambiguity, the user cannot comprehend the link between input and outcome.	The accuracy of LDA, MLE, and BPNN was 100%.	[61]
15	SVD, PCA, SVM	SVD and PCA: decompose 152 features into two principal components. SVM: analyze the obstacle data from the impedimetric biosensor.	Gaussian RBF kernel with high correctness.	The dataset for the default kernel settings is not linearly divisible. RBF^o)^ kernel suffers from an overfitting problem.	SVM achieved an accuracy of 95±4% in cross‐validation and test sample prediction.	[62]
16	DT, RF, NB, ANN, SVM, LSSVM	Learn the features of the training samples to predict the concentration of nitrate in liquid samples with a wide pH range (3.5–8.5).	It is possible to improve the accuracy and reliability of the response.	—	SVM outperformed other ML methods. SVM predicted the nitrate concentration of plant‐ and bacterial‐based enzymes with R^2^ = 0.97 and 0.96.	[37h]

^a)^ BPNN: back propagation neural network

^b)^ R: coefficient of correlation

^c)^ RSD: relative standard deviation

^d)^ Clay/AuNPs/AChE: clay mineral/gold nanoparticles/acetylcholinesterase

^e)^ PLS‐DA: partial least squares discriminant analysis

^f)^ SISSO: sure independence screening and sparsifying operator

^g)^ BES: bio‐electrochemical sensor

^h)^ VFA: volatile fatty acid

^i)^ SVD: singular value decomposition

^j)^ PCR: principal component regression

^k)^ MTX: methotrexate

^l)^ EIS: electrochemical impedance spectroscopy

^m)^ SOM: self‐organized maps

^n)^ LDA: a linear discriminant analysis

^o)^ RBF: radial basis function

As seen from Table 4, the purpose of ML in EC biosensors is mainly to classify, predict, and discriminate the influencing factors, which are discussed in the following sections.

#### 3.1.1 Classification

ML algorithms automatically learn complex feature relationships from raw signals generated by EC biosensors, thus avoiding the subjective bias and workload associated with manual feature selection. In addition, ML algorithms deal with the issues related to nonlinear decision boundaries, and thus provide more accurate classification for EC biosensors. Therefore, ML algorithms assist EC biosensors in classifying substances in a fast and accurate way.

**Figure 5:**
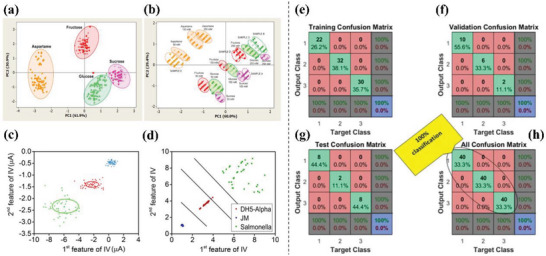
Based on a) the type of sugar and b) the content of several common beverages, sugar was categorized. Reproduced with permission.^[^ ^51^ ^]^ Copyright 2022, Springer. c) Prediction of three bacterial samples (*E. coli* DH5‐α, JM 109, and Salmonella are depicted in red, blue, and green, respectively) using the MLE model and d) LDA clustering analysis of three bacteria. Reproduced with permission.^[^ ^61^ ^]^ Copyright 2018, Springer. Classification of bacteria with BPNN: e) 70% training, f) 15% validation, g) 15% test data, h) 40 samples of each kind of bacteria correctly classified. Reproduced with permission.^[^ ^61^ ^]^ Copyright 2018, Springer.

Sugar is an additive in beverage products and excessive consumption of sugar leads to an increase in various diseases. Therefore, it is necessary to classify and detect the sugar in drinks. Umar et al.^[^ ^51^ ^]^ used a whole‐cell immobilized amperometric biosensor to determine the sugar content in bottled beverages. PCA was used to calculate the proportion of errors by comparing the reported concentration values indicated on the sample package composition with the concentration data discovered by measurements. **Figure** **5**a,b highlight the difference between natural sugars and artificial sweeteners. The narrower clustering pattern of natural sugars indicates that the detection process of natural sugars is stable, and PCA distinguishes their type and concentration. In contrast, the data generated by each sweetener concentration has a high degree of overlap, and thus it was difficult to classify both sweeteners and their concentrations measured by the sensor. Therefore, PCA was used to reduce the irrelevant data generated in measurements, so that the data is classified and clustered.

In addition, ML algorithms are used to classify bacteria. Classification of bacteria is important in many practical applications, such as the food industry. Ali et al.^[^ ^61^ ^]^ proposed a new impedance‐based biosensor that easily and quickly detects three different types of bacteria, including *Salmonella typhimurium*, and the *Escherichia coli* strains JM109 and DH5‐α. The biosensor's capability was evaluated using three kinds of algorithms, including MLE, LDA, and BPNN, and accuracy was used as the evaluation parameter. As shown in Figure 5c, the MLE classifier's overall accuracy is 100%. In the case of LDA, except for a few samples of these bacterial groups at the edges of the linear discriminant plane, all test samples clustered well along the two hyperplanes (Figure 5d). The overall accuracy of LDA reaches 100%, indicating an accurate distinction of bacterial types. Moreover, nonlinear BPNN was also applied to a given dataset classification problem. A total of 120 data vectors were employed, of which 84 were used for training and 36 were equally split between the test and validation datasets (Figure 5e–g). In training, testing, and cross‐validation, the BPNN achieved 100% accuracy, accurately classifying every bacterial sample (Figure 5h). The accuracy of MLE, LDA, and BPNN for bacterial classification reached 100%, demonstrating that these ML algorithms achieved good results in classifying different bacterial classes and the scheme is simple, fast, accurate, and economical.

The ML algorithms used in the above studies include PCA, MLE, LDA, and BPNN. PCA helps remove redundant information and noise and find the main features in the data. MLE finds the most likely classification result according to the probability distribution of data, providing a more flexible classification method. LDA finds the optimal projection direction, maximizes the distance between different categories, and minimizes the variance within the same category, thus improving the accuracy of classification. BPNN is a powerful nonlinear model with strong fitting ability. It learns complex feature relationships through multi‐layer neural networks and automatically adjusts network weights so that the model adapts to different complex classification tasks. These algorithms assist EC biosensors in classification tasks.

#### 3.1.2 Prediction

There are multiple influencing factors involved in EC biosensors, and ML algorithms automatically synthesize multiple features for prediction and capture the comprehensive influence of multiple factors. Thus, ML makes it possible to forecast the concentration of compounds detected by EC biosensors more quickly and accurately. The algorithms used for prediction possess the advantages of linear or nonlinear signal stimulation, real‐time operation, and rapid calculations.

**Figure 6:**
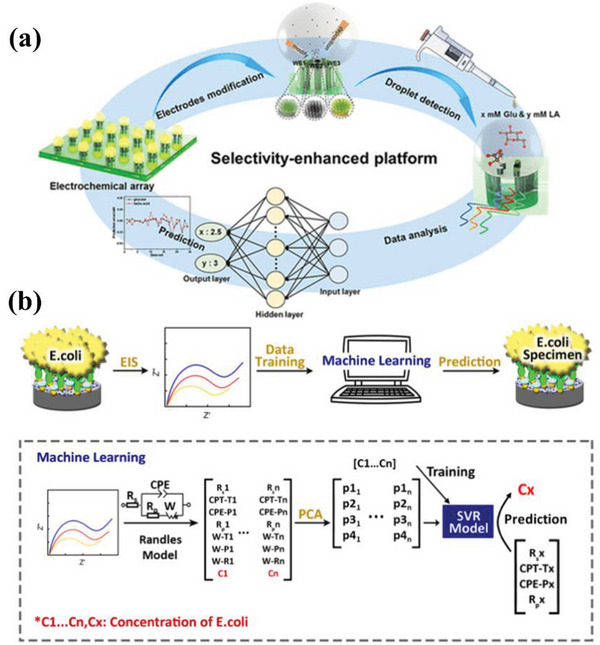
a) Illustration of highly specific non‐enzymatic electrochemical sensing with BPNN. Reproduced with permission.^[^ ^38b^ ^]^ Copyright 2022, ACS. b) A schematic representation of an EIS biosensor system based on ML for *E. coli* detection. Reproduced with permission.^[^ ^58^ ^]^ Copyright 2020, IOP.

To overcome the overlapping of glucose and lactate oxidation peaks and enhance the selectivity of non‐enzymatic electrochemical detection, Zhou et al.^[^ ^38b^ ^]^ introduced BPNN into non‐enzymatic electrochemical biosensing (**Figure** **6**a). By analyzing the chronoamperometry results of multiple EC biosensors, the non‐enzymatic sensors using BPNN could achieve high sensitivity and wide‐range detection of glucose and lactate, with an R^2^ value of 0.9997 and a relative standard deviation of less than 6.5%. The results show that BPNN could help identify and predict glucose and lactate concentrations. It can be seen that BPNN has been used to perform both classification and prediction tasks. The reason is that its multi‐layered structure and backpropagation algorithms allow it to learn complex features from input data and make classifications or predictions based on those features. For classification tasks, BPNN judges the category of a new sample by learning the relationship between input data and the corresponding labels. For prediction tasks, it predicts continuous values by learning the relationship between input data and actual output values.

Additionally, ML techniques have been applied to enhance the precision of bacterial concentration determination. Xu et al.^[^ ^58^ ^]^ employed an ML‐based electrochemical impedance spectroscopy (EIS) biosensor to detect *E. coli* (Figure 6b). The goal was to use EIS data to automatically synthesize numerous impedance parameters into a recognition machine that determines bacterial concentrations. To automatically create quantitative correlations between multiple impedimetric parameters and bacterial concentrations, PCA was used to extract impedance parameters from EIS data recorded at various bacterial concentrations. Subsequently, the first four main components (p1, p2, p3, p4) were kept and fed into the SVR, with the concentration of *E. coli* serving as the model output. The average prediction error (*n* = 10) reached 1.52 ± 0.136%, revealing that the ML model accurately determines *E. coli* concentrations and has advantages in adaptability, automation, and accuracy. ML‐based EIS biosensors exhibit a self‐learning capability and hence are more adaptable to a variety of sensor designs for molecular and cellular detection. Furthermore, if more dimensions of EIS data are studied, the detection accuracy may be further enhanced.

The ML algorithms used in the above studies include BPNN, PCA, and SVR. BPNN automatically learns complex nonlinear relationships and is suitable for processing nonlinear EC biosensor data. PCA be used to reduce the dimensionality of data, remove redundant information and noise in data, and improve the efficiency and generalization ability of prediction models. SVR learns complex nonlinear patterns through kernel function techniques to provide high prediction accuracy. These algorithms improve the prediction ability of EC biosensors.

#### 3.1.3 Discriminating Influencing Factors

**Figure 7:**
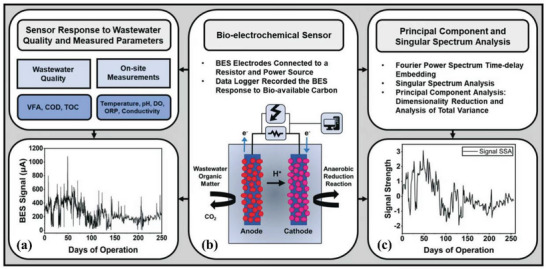
Overview of the BES case study for field applications. a) From June 20, 2019, to February 22, 2020, BES response signals (also known as MET, or microbiological electron transfer) were recorded, and within the same time frame, field measurements were taken to compare them to the BES signals. b) After being transmitted over an Internet connection via the HTTP or MQTT network protocol, data were stored on a cloud‐based data server and, for redundancy, added to a local SD card at the controller. c) To assess the BES signals in reaction to environmental conditions, PCA, KPCA, and SSA were applied. Reproduced with permission.^[^ ^55^ ^]^ Copyright 2022, the Royal Society of Chemistry.

ML algorithms automatically learn the correlation between multiple influencing factors, find complex causal relationships, and assist factor discrimination. To determine what and how certain influencing elements impact EC biosensors, ML algorithms have been employed in reports. For instance, Emaminejad et al.^[^ ^55^ ^]^ have used sophisticated data analysis and microbiological approaches to measure the sensitivity of a bio‐electrochemical sensor (BES) deployed in the major effluent channel of a water resource recovery facility (**Figure** **7**). PCA was used as a dimensionality reduction approach to expose the pattern and direction of the highest variance in data and to demonstrate the effect of BES signal variance on observed environmental factors. However, PCA is built on linear assumptions, which is inapplicable to biological wastewater treatment systems with inherently nonlinear features. Therefore, kernel PCA (KPCA) was used to interpret the data's nonlinearity. In addition, singular spectrum analysis (SSA) was conducted to recognize the system structure in biosensor signal responses and compare it to a dissolved oxygen probe mounted in an active sludge tank near the BES probe.

Results show that PCA revealed substantial differences in sensor response signal behavior between warm and cold months, and no significant linear effects of any of the examined factors on total signal variance were discovered during the cold weather runs. KPCA confirmed the nonlinearity of the cold weather data. The signal was impacted by seasonal and monthly cycle patterns, which may be attributable to the influence of rainfall events and seasonal temperature fluctuations, according to SSA. These algorithms possess the capability to rapidly and distinctly explicate the long‐term carbon monitoring potential of BESs under diverse environmental circumstances. Therefore, ML methods are used to investigate the linear or nonlinear interaction between environmental conditions and biosensor signals to improve the sensing capability of biosensors.

ML algorithms such as PCA, KPCA, and SSA were used to discriminate influencing factors. PCA is suitable for reducing and removing data noise, KPCA is suitable for dealing with nonlinear relations, and SSA is suitable for dealing with time series data. These algorithms help to identify the key influencing factors and improve the accuracy and efficiency of identifying influencing factors.

### 3.2 Applications of Machine Learning in Microbial Fuel Cells

With the help of immobilized cell populations, MFCs employ bacteria as catalysts to oxidize both organic and inorganic materials, dramatically lowering the barrier to electron transfer in biofilms and solid electrodes to achieve the necessary power production capacity.^[^ ^27^ ^]^

Due to the sensitivity of MFCs to environmental factors, the mathematical modeling of MFC models is difficult.^[^ ^63^ ^]^ MFCs are a complex nonlinear process that requires a strategy nonlinearly controlled to obtain the most favorable results. ML helps reduce computational and modeling costs, saves time, and is more efficient than manual methods previously used.^[^ ^63^ ^]^ **Table** **5** shows the applications of different ML algorithms in MFCs from 2018 to 2022 (in descending order of publication date) from 25 research papers.

**Table 5:** Applications of ML in MFCs.

#	Algorithms	Application Scenarios	Advantages	Disadvantages	Results	Ref.
1	CNN, JSCNN, LR^a)^, ANN, SVR, CART^b)^, KNN	Predict power generation from PMFCs.	The computation of the hybrid JSCNN model is fast.	The accuracy of LR, ANN, SVR, CART, and KNN is low.	The MAPE^c)^ was 11.00% for PMFC data containing wolfsbane and 11.88% for PMFC data containing narrow‐leaved balsam.	[64]
2	PCA	Examine how the mixing ratio affects the hydrolytic breakdown and energy recovery of SPW^d)^.	Accurate and simple.	—	Throughout the procedure, the composition of dissolved organic matter was significantly impacted by various SPW mixing ratios.	[65]
3	KNN, RBF	Identify compounds based on voltage patterns.	RBF's improvement on KNN may better determine the confidence level.	KNN cannot output unknown confidence levels.	RBF, and consistent interpreters classified gasoline, urea, and fertilizer with 100%, 88%, and 94.5% accuracy.	[66]
4	PCA	Compare the electroactive capacity of microbial communities.	Select the optimal combination of parameters.	—	Blue and green lakes with the lowest power output of concentrated cultures.	[67]
5	SVR	Record the MFC voltage and run the SVR.	The high correlation between input (characteristics) and output (COD values).	Ignore individual COD values to produce voltage distributions with different peak heights.	The device accurately measured COD in natural pond water samples (R^2^ = 0.94).	[18]
6	Augmented K‐means clustering	Determine the number of cycles for the stable CV curve. Predict the duration of the stable power output.	Overcome the disadvantages of k‐means clustering.	The parameters are set with a bound on the associated minimum and maximum input values.	An excellent estimate of the CV cycles required to obtain a stable voltage‐current curve was obtained.	[27]
7	ANN	Obtain the best model for predicting the MFC power output.	The power output of MFCs is swiftly and precisely predicted by ANN.	ANN cannot provide additional outputs.	The R^2^ values for SCG^e)^ and time series were 0.98802 and 0.99115, respectively.	[37d]
8	PCA	Evaluate the relationship between different samples.	The relationship between different populations was successfully evaluated.	—	The microbial structure of UMFCs^f)^ was similar to that of the methane bioreactor.	[68]
9	NARX^g)^	Predict the electrical output of MFCs.	Easy to implement, with the ability to switch modes.	Performance is vulnerable to initial values.	R: 0.99978 (training), 0.99988 (validation), 0.99994 (test), and 0.9998 (whole data set).	[38a]
10	PCA	Determine the factors that affect MFC voltage and power density.	PCA accurately determines the factors.	—	These factors include volume, hydraulic retention period, COD loading rate, COD removal, and internal resistance.	[69]
11	PCA	Investigate the effect of external electrical impedance.	PCA shows how external resistance affects the microbial population in the anode chamber.	—	The external resistance induced changes in substrate removal and power generation, which in turn affected the microbial community.	[70]
12	ANN	Simulate the impact of flow rate on the output power of a ceramic MFC made from human urine.	QN^h)^, LM^i)^, and CG^j)^ accurately simulate power prediction.	It is impossible to predict in advance the ideal number of neurons for the buried layer.	The LM algorithm had the highest accuracy (R = 95%) and the shortest convergence time (7.8s).	[12]
13	PCA	Investigate the effects of Zn addition and circuit patterns on different factors.	The interaction between different factors is successfully analyzed.	—	Significant accumulation of antibiotics and zinc and circuit patterns significantly influenced the distribution characteristics of ARGs^k)^ and bacterial communities.	[71]
14	RBA^l)^, WkNN^m)^, GMA^n)^, MWA^o)^, HT^2^S^p)^	Control integration in the MMB^q)^ control strategy.	WkNN is able to solve nonlinear problems.	No other multivariate dynamic models are available.	Using WkNN as the model switching method for MMB reduces the average setup time by about 65% compared to the single linear model controller.	[39]
15	PCA	Evaluate the formation of biofilms on the anode surface of MFCs operated.	Successfully analyze significant differences in anode biofilm maturation in different MFCs.	—	The MFC performance is significantly higher for dynamic adjustment of external resistance, but the operational stability is relatively low.	[72]
16	ANN	Adjust the optimal conditions for the proper operation of MFCs.	Provide adaptive solutions and re‐estimation of model parameters in a relatively simple form.	The dataset must contain at least 100 input/output patterns.	The internal resistance of the system was reduced to 1.63 × 10^3^ Ω cm^2^. This resulted in a high power density of 8314 mW m^−2^.	[73]
17	PCA	Describe how the microbial community of MFCs is affected by the S: N ratio.	Effective and simple.	—	The first and second principal components were responsible for 59.59% and 33.04% of the total variance, respectively.	[74]
18	PLS, KNN, RF, NNET^r)^	Predict resistance and resilience.	NNET: fully approximate complex nonlinear relationships. RF and KNN: no need to select a feature.	The inadequate sample size for model development and evaluation.	The classification accuracy of the resistance and elasticity classes corresponding to the risk of deactivation was 70.47 ± 15.88% and 65.33 ± 19.71%.	[22]
19	PCA	Examine how toxins and microorganisms that produce electricity are related.	Accurate and simple.	—	The microbial community shifted from left to right with increasing concentrations of Cu (II) and 2,4‐dichlorophenol.	[75]
20	ANN	Estimate the voltage of each MFC.	ANN is developed very quickly and easily.	Time‐consuming and does not provide additional output.	*R* = 0.99662.	[76]
21	Recursive Bayesian, KNN, Hotelling's T^2^	The suggested control scheme's efficacy is validated, and switching approaches are contrasted.	KNN is simple and easy to implement.	—	The stabilization time of the MMB control strategy is reduced by ≈47.3% compared to the single‐model linear proportional‐integral controller.	[77]
22	PCA	Investigate the effects of different temperatures. Analyze the microbial community structure of the anode biofilm.	PCA successfully visualizes the structure of the anode biofilm community of MFCs.	—	A clear separation between the inoculum pretreated at 4 °C and 10 °C, and the inoculum pretreated at low temperatures forms the bacterial community structure.	[78]
23	GLMNET^s)^, RF, XGBOOST^t)^, NNET, KNN, SVM	Train input variables and evaluate their ability to predict feed substrates from genomic datasets.	Feed substrates are successfully predicted. The specificity of the MFC‐based biosensor signal is improved.	More samples and input features need to be considered.	The model developed by the NNET algorithm had the highest accuracy (93 ± 6%), corresponding to a kappa value of 0.87 ± 0.10.	[20]
24	ANN, RVM, ELM^u)^, GPR^v)^, SVM	SVM is used for classification and other algorithms are used for regression.	SVM works faster and better in high‐dimensional space. RVM: strong sparsity and generalization ability, short testing time, suitable for online detection.	ANN: slow convergence and local miniaturization.	R^2^ for offline performance model evaluation: ELM (0.9998)>ANN (0.9983)>RVM (0.9951)>GPR (0.9664)	[79]
25	PCA	Use dairy feed water with different characteristics and analyze using PCA.	PCA determines the possible reduction in the number of data dimensions generated when manipulating MFCs.	—	The eigenvalue of PC1 was 2.84 with a variance of 35.54%, the eigenvalue of PC2 was 2.04 with a variance of 25.46%, and the eigenvalue of PC3 was 1.56 with a variance of 19.51%.	[80]

^a)^ LR: linear regression

^b)^ CART: classification and regression tree

^c)^ MAPE: mean absolute percentage error

^d)^ SPW: solid potato waste

^e)^ SCG: scaled conjugate gradient

^f)^ UMFCs: up‐flow air‐cathode chamber microbial fuel cells

^g)^ NARX: nonlinear autoregressive networks with exogenous inputs

^h)^ QN: Quasi‐Newton

^i)^ LM: Levenberg‐Marquardt

^j)^ CG: Conjugate Gradient

^k)^ ARGs: antibiotic resistance genes

^l)^ RBA: recursive Bayesian approach

^m)^ WkNN: weighted k‐nearest neighbor

^n)^ GMA: gap metric approach

^o)^ MWA: multi‐model weighting algorithm

^p)^ HT^2^S: Hotelling T‐squared strategy

^q)^ MMB: multiple model‐based control

^r)^ NNET: neural network

^s)^ GLMNET: logistic regression multiclass

^t)^ XGBOOST: scalable tree boosting system

^u)^ ELM: extreme learning machine

^v)^ GPR: Gaussian process regression

As seen from Table 5, the purpose of ML in MFCs is mainly to classify, predict, and discriminate the influencing factors, which are discussed in the following sections.

#### 3.2.1 Classification

MFCs involve the relationship between multiple complex factors, and ML algorithms automatically learn and capture these complex relationships to improve the accuracy of classification tasks.

By exposing terrestrial soil‐based microbial fuel cells (tMFCs) to gasoline, petroleum, 2,4‐dinitrotoluene, fertilizer, and urea, Barbato et al.^[^ ^66^ ^]^ created a sensor technique for molecules suggestive of anthropological compounds. RBF and KNN were trained to detect chemicals based on voltage patterns. KNN is one of the simplest ML algorithms,^[^ ^81^ ^]^ and RBF improves on it by using the distance between the input and the training data set to evaluate confidence and provide “unknown” confidence.^[^ ^82^ ^]^ For KNN, the classification of the test input is defined by its K nearest neighbors in the training input, with the majority of the K nearest neighbors having the same classification as the test input. Because the KNN approach fires all neurons, it cannot produce an “unknown” confidence level. The RBF approach is identical to KNN, except that the neuron only fires if its internal weight is within one unit of the input parameter. Compared with KNN, RBF classifies anthropological compounds more effectively. Specifically, RBF was able to categorize gasoline, urea, and fertilizer with 100%, 88%, and 94.5% accuracy, demonstrating that tMFCs could be utilized as biosensors for environmental monitoring.

The advantage of ML algorithms in MFC classification tasks is high accuracy and effectiveness. Compared with a simple KNN algorithm, RBF classifies samples containing man‐made compounds more effectively, thus providing a reliable classification solution for the application of MFC in environmental monitoring and other fields.

#### 3.2.2 Prediction

**Figure 8:**
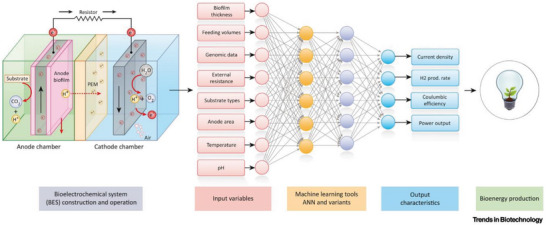
ML‐driven MFC system. Reproduced with permission.^[^ ^83^ ^]^ Copyright 2022, Elsevier.

The performance of MFCs is affected by a variety of factors, including temperature, pH, substrate type, etc., and ML algorithms consider multiple factors at the same time and build complex predictive models. For example, bioelectrochemical reaction rates in MFCs were reported to be improved by introducing self‐calibrating and automatically optimizing ML tools such as ANN. ANN algorithms allow easier model creation, which do not require predefined knowledge to create a new model but have the disadvantage of not providing additional outputs in addition to those being trained.^[^ ^63^ ^]^ **Figure** **8** illustrates the various inputs and outputs of an MFC coupled with ANNs (nodes, layers, and networks). These ANNs were used to predict performance more accurately by simulating the weights of important parameters for forward and backward propagation through the layers.^[^ ^83^ ^]^ With the help of neural networks, it is also possible to filter key performance indicators and determine their sensitivity.^[^ ^83^ ^]^

**Figure 9:**
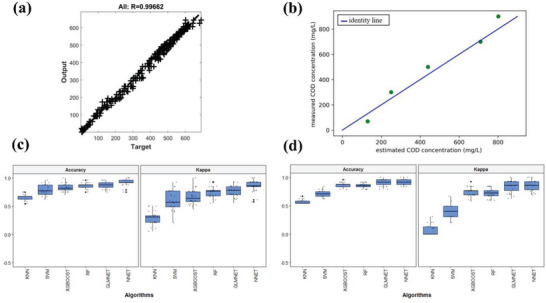
a) Regression plot comparing network results to the objectives of the entire dataset. Reproduced with permission.^[^ ^76^ ^]^ Copyright 2019, Elsevier. b) The connection between the observed and estimated COD concentrations is shown by the integrated system's sensing data (R^2^ = 0.94). Reproduced with permission.^[^ ^18^ ^]^ Copyright 2021, IEEE. Phyla dataset c) and family dataset d) combined with accuracy and kappa metrics of several algorithms. Reproduced with permission.^[^ ^20^ ^]^ Copyright 2019, Elsevier.

The following is an example of the specific application of ANN in MFCs. In order to predict the voltage output of MFCs in polarization tests, Tsompanas et al.^[^ ^76^ ^]^ developed an ANN with a 4‐10‐1 topology. The reason for using ANN is that ANN is ideal for studying complex MFC systems because it is not necessary to know the detailed rules of the control system. After one training, the network displayed a high correlation coefficient (R) of 0.99662 for the complete dataset (**Figure** **9**a), indicating its exceptional proficiency in accurately and promptly predicting the voltage output of MFCs. In future research, a time component is encouraged to be introduced to ANN to predict MFC output as a time series.

In addition to ANN, other ML algorithms have also been used for prediction. Shabani et al. proposed an energy‐autonomous water quality monitoring device with a single MFC as its sensory input and the only power source for computing chemical oxygen demand (COD).^[^ ^18^ ^]^ To strike a good balance between high accuracy and low execution time, an SVR with an RBF core was selected to find the relationship between MFC output voltage and COD. The geometric properties of MFC voltage distribution were sent into the SVR as input. A low‐power microcontroller that records the MFC voltage and powers the SVR was driven by the power produced by MFCs. The device was capable of precisely detecting COD in water samples from natural ponds, with R^2^ = 0.94 (Figure 9b). In the experiments, a large range of COD (70–900 mg L^−1^) was considered. Training the algorithm in a smaller range results in higher accuracy in that range.

In reports, the ML algorithm predicts not only COD but also feed substrate. To test whether it was possible to identify feed substrates from the ensuing microbial communities, Cai et al.^[^ ^20^ ^]^ gathered 69 samples of three distinct substrate types (acetate, glucose, and wastewater) from various laboratory conditions. The capacity of neural network (NNET), scalable tree boosting system (XGBOOST), logistic regression multiclass (GLMNET), RF, KNN, and SVM to predict feed substrates from genomic datasets were trained and assessed. The identification of suitable data inputs and the selection of appropriate ML algorithms provide a direct link between substrate groupings and genomic data without the need for additional information such as operating conditions and currents, making this approach more broadly applicable to systems with mixed microbial communities. The model built by the NNET algorithm showed the highest accuracy. The accuracy and *kappa* values of NNET trained on the dataset with phyla classification were 93 ± 6% and 0.87 ± 0.10, respectively (Figure 9c). The accuracy and *kappa* values of NNET trained on the dataset with family classification were 92 ± 5% and 0.86 ± 0.09, respectively (Figure 9d). These findings reveal a novel use of ML approaches with significant practical implications in the field of biotechnology for feed substrate prediction and MFC‐based biosensor signal specificity enhancement.

Through the above research, it can be concluded that the advantages of ML algorithms in MFC prediction tasks mainly include: high precision and fast execution, suitable for complex systems, and prediction of a variety of parameters. ANN could accurately and quickly predict the voltage output of MFC, SVR found a good balance between high accuracy and low execution time to predict COD, and NNET excelled at predicting substrates. The applications of ML algorithms in MFC have important practical values and bring significant advantages for substrate prediction and MFC‐based biosensor signal enhancement.

#### 3.2.3 Discriminating Influencing Factors

PCA can be used not only for classification but also to identify influencing factors. In classification, PCA is used to reduce dimension. By selecting the appropriate number of principal components, the accuracy, and efficiency of classification are improved by retaining high data information while reducing the number of features. For the identification of influencing factors, the relevant variables are transformed into a set of unrelated principal components by PCA. These principal components reflect the different influencing factors in data and are ordered by the magnitude of their explanatory variance. Therefore, by analyzing the load of principal components (i.e., the relationship between principal components and original variables), it is possible to determine which variable contributes the most to the principal component.

**Figure 10:**
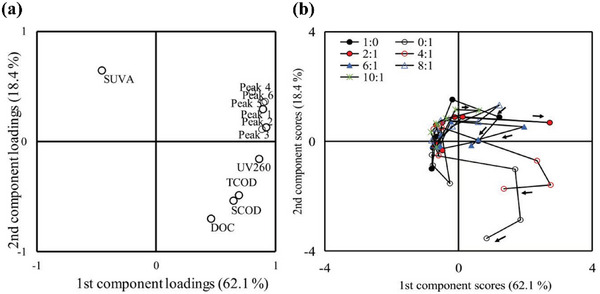
PCA was performed on data from TCOD, SCOD, DOC, UV260, SUVA, and 5 EEM peak intensity changes of MFCs with various SPW and WAS mixing ratios. a) The component loading plots. b) The component score plots. Reproduced with permission.^[^ ^65^ ^]^ Copyright 2022, Elsevier.

The following is a study in which PCA was used to identify influencing factors. Du et al.^[^ ^65^ ^]^ introduced the synergistic effect of potato solid waste (SPW) and waste‐activated sludge (WAS) to improve the waste conversion capacity of MFCs. PCA was used to successfully examine the influence of the mixing ratio on the hydrolytic breakdown and energy recovery of SPW. The higher loadings of peaks 1–6 and UV260 in the same direction as PC1 in **Figure** **10**a indicate that SPW and WAS were effectively hydrolyzed. SUVA and DOC loadings as PC2 in the opposite direction are rather high. This might be due to the varying rates of the humic compound and other dissolved organic matter breakdown in MFC anode dissolution solutions. For PC1 in Figure 10b, except for the fractions with mixing ratios of 2:1 and 10:1, the scores of all fractions increased positively and then reversed, demonstrating a rise in the reduction in tryptophan‐ and tyrosine‐like amino acids, aromatic proteins, and humic compounds with time. Except for PC2 with mixing ratios of 2:1 and 10:1, which increased positively with time, the rest of PC2 increased positively and then reversed to positive, and PC2 with the mixing ratio of 0:1 scored the highest. Dissolved organic matter in WAS was more easily degraded, and the relative content of humus‐like substances in dissolved organic matter was higher. The results show that the mixture ratio of SPW and WAS had a significant effect on the composition of dissolved organic matter. Therefore, PCA helps better understand the association between total chemical oxygen demand, soluble chemical oxygen demand, dissolved organic carbon, ultraviolet absorbance at the wavelength of 260 nm, specific ultraviolet absorbance, and primary fluorescence peak intensity of each sample during MFC operation. PCA is further used to analyze the factors that affect MFCs and provide a reference for improving MFC performance.

#### 3.2.4 Switching Models

**Figure 11:**
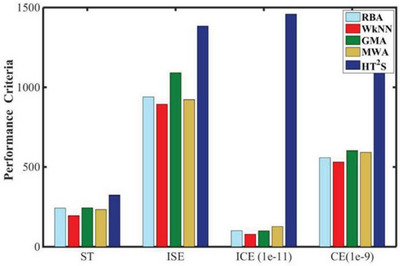
Average performance criteria for MMB controller's multiple switching mechanisms (ST: stability time; ISE: integral square of error; ICE: integral control command; CE: control command). Reproduced with permission.^[^ ^39^ ^]^ Copyright 2020, Elsevier.

ML has been reported to switch MFC models from algorithms including RBA, WkNN, GMA, etc. For instance, Yewale et al.^[^ ^39^ ^]^ proposed an MMB controller strategy to solve the nonlinearity problem in CMFC and implemented it on the developed MIMO system. For MMB controllers, the model‐switching approach needs to work precisely on the overlap of multiple subspaces created by decomposing operational regimes into local regimes. Therefore, weighted methods, including recursive Bayesian methods (RBA), WkNN, GMA, MWA, and HT^2^S, were used for MMB controller integration to combine many controllers into a single global controller.^[^ ^84^ ^]^ Besides, the gap metric technique was used to break the nonlinear portion of CMFC into numerous linear regions. As shown in **Figure** **11**, WkNN outperformed other approaches. The average stabilization time of the MMB controller using WkNN was lowered by roughly 65% when compared to the single linear model controller, and thus the nonlinear problem of CMFC was solved successfully. In short, ML algorithms such as WkNN demonstrate an outstanding ability to deal with complex nonlinear problems of MFCs in switching models more efficiently, accurately, and flexibly, affording strong support for the development of MFC control strategies.

### 3.3 Applications of Machine Learning in Microbial Electrosynthesis

Only several works applied ML to microbial electrosynthesis and mainly concentrated on experimental data analysis. Currently, an unsupervised learning algorithm, hierarchical clustering, has been introduced to microbial electrosynthesis. The principle of hierarchical clustering is that given a set of data points, the output is a binary tree (tree graph). Its leaves are data points, its internal nodes represent nested clusters of varying sizes, and the tree organizes these clusters in a hierarchy that hopefully aligns with the intuitive organization of real‐world data.^[^ ^85^ ^]^ Here is an example.

**Figure 12:**
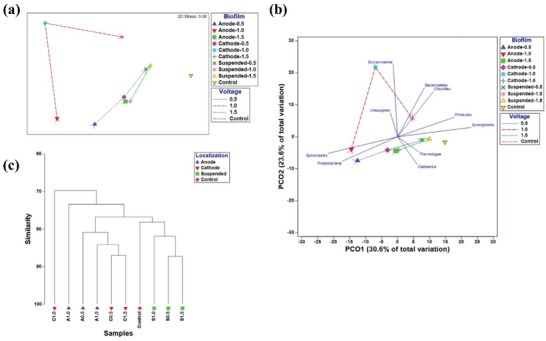
Anode‐A, cathode‐C, and suspension‐S biofilm communities from each MES‐AD (0.5, 1.0, and 1.5 V) and control biofilms were used as the basis for a systematic cluster analysis of nMDS, PCO, and BrayeCurtis indices. a) The nMDS plot. b) The PCO plot. c) The hierarchical cluster plot. Reproduced with permission.^[^ ^47^ ^]^ Copyright 2016, BMC.

To improve the methane production rate of anaerobic digestion microbial electrosynthesis, Flores‐Rodriguez and Min^[^ ^47^ ^]^ examined the dispersion of microbial populations at varied voltages (0.5, 1.0, and 1.5 V) to find the optimal voltage for the enrichment of specific microbial communities. It was found that methane generation is more advantageous at 1.0 V. Then, all samples were submitted to hierarchical clustering based on the Braye Curtis index, principal component vertical coordinate (PCO), and non‐metric multidimensional scaling (nMDS) (**Figure** **12**). Significant variations between samples were visible in the nMDS and PCO plots (Anosim, *P* = 0.009, *R* = 0.377) (Figure 12a,b). The findings of the nMDS and PCO plots are supported by the hierarchical clustering plot (Figure 12c). The C1.0 biofilm aggregated independently of other biofilms, indicating the distinct enrichment of microbial biofilms generated by the applied voltage. In this work, hierarchical clustering assists in undertaking a non‐quantitative multidimensional analysis to discover systematic differences between samples.

Hierarchical clustering in microbial electrosynthesis helps to analyze the similarities and differences of microbial communities, reveal systematic changes, infer biological reactions, and optimize reaction conditions. The application of hierarchical clustering in microbial electrosynthesis provides an effective analytical tool for researchers to understand the complex community structure and influencing factors in microbial electrosynthesis.

## 4 Summary and Outlook

Bioelectrocatalysis is a multi‐factors involved complicated system that is challenging to study simply through artificial experiments. The ability to cope with nonlinear issues, classification, prediction, identification of influencing factors, optimization of operating conditions, and other operations, reflects the originality and advantage of ML applications in bioelectrocatalysis. Despite these achievements in ML‐assisted bioelectrocatalysis research, there are still many challenges, problems, and unexplored potential research fields.

1. There are a few relevant databases, which should be established in the future to facilitate access to large amounts of bioelectrocatalysis data. Moreover, the data used are mainly collected manually from experiments or literature. ML could also assist in the data collection process, especially through popular large language models. In addition, existing data could be utilized for more efficient algorithmic tuning, such as employing data augmentation techniques to expand the size of datasets. Data scarcity can also be mitigated through domain knowledge sharing and collaboration, which avoids redundant data collection, thereby reducing costs and resource wastage.
2. Specifically for EC biosensors, although many applications such as food evaluation and medical diagnostic analysis have benefitted from ML, the hurdles of stability, sensitivity, dependability, and simplicity for practical use and commercialization require the involvement of ML more broadly and deeply.
3. MFCs can be used for wastewater treatment, desalination, water quality testing, green power supply, etc. Its performance is heavily influenced by complex factors including substrates in the anode chamber, anode and cathode materials, electrolytes, operating temperature, and the situation of microorganisms.^[^ ^63^ ^]^ These multi‐dimensional data could provide a great opportunity for training and developing bioelectrocatalysis‐fitted ML algorithms, which may potentially solve the low electrical power generation efficiency issue that has long restricted the use of MFCs.
4. ANN is one of the most used ML methods in research of bioelectrocatalysis, but there are still many problems, such as long training time, the requirement of large amounts of training data, lack of incremental learning ability, not quite suitable for high‐precision computing, etc. The study on ANN is thus still crucial with emphasis on some practical issues, including the creation of a universal neural network processor or implementation of a huge number of dynamic neural‐free linkages.
5. The applications of ML in bioelectrocatalysis are limited to EC biosensors and MFCs. Its exploration in bioelectrosynthesis and other fields deserves more attention and effort.

ML has great potential to be useful in bioelectrocatalysis for clean energy production and organic waste/environmental pollutant treatment. It optimizes reaction conditions and increases bioelectrocatalysis efficiency. Besides, it is employed in data/signal processing and classification. Deeper rules and patterns may be mined using ML, which can help to speed the development of bioelectrocatalysis technology. Such an interdisciplinary research direction is showing great potential to change the rules of the traditional research paradigm of bioelectrocatalysis.

## Conflict of Interest

The authors declare no conflict of interest.
